# 13-year nationwide cohort study of chronic kidney disease risk among treatment-naïve patients with chronic hepatitis B in Taiwan

**DOI:** 10.1186/s12882-015-0106-5

**Published:** 2015-07-22

**Authors:** Yi-Chun Chen, Yu-Chieh Su, Chung-Yi Li, Shih-Kai Hung

**Affiliations:** Division of Nephrology, Department of Internal Medicine, Dalin Tzu Chi Hospital, Buddhist Tzu Chi Medical Foundation, Chiayi, No. 2, Minsheng Rd., Dalin Township, Chiayi, County 622 Taiwan; School of Medicine, Tzu Chi University, Hualien, Taiwan; Division of Hematology-Oncology, Department of Internal Medicine, Dalin Tzu Chi Hospital, Buddhist Tzu Chi Medical Foundation, Chiayi, Taiwan; Department and Graduate Institute of Public Health, College of Medicine, National Cheng Hung University, Tainan, Taiwan; Department of Public Health, College of Public Health, China Medical University, Taichung, Taiwan; Department of Radiation Oncology, Dalin Tzu Chi Hospital, Buddhist Tzu Chi Medical Foundation, Chiayi, Taiwan

**Keywords:** HBV infection, Chronic kidney disease, Cohort study, Taiwan National Health Insurance Research Database, Competing mortality

## Abstract

**Background:**

Chronic hepatitis B virus (HBV) infection and chronic kidney disease (CKD) have high prevalences in Taiwan and worldwide. However, the association of untreated chronic hepatitis B virus (HBV) infection with chronic kidney disease (CKD) remains unclear.

**Methods:**

This cohort study used claims data in the Taiwan National Health Insurance Research Database in 1996–2010, in which all diseases were classified by ICD-9-CM codes. We identified 17796 adults who had chronic HBV infection and did not take nucleos(t)ide analogues from 1998 to 2010 and also randomly selected 71184 matched controls without HBV in the same dataset. Cumulative incidences and adjusted hazard ratio (aHR) of incident CKD were evaluated through the end of 2010 after adjusting for competing mortality.

**Results:**

The risk of CKD was significantly higher in the HBV cohort (13-year cumulative incidence, 6.2 %; 95 % confidence interval [CI], 5.4–7.1 %) than in the non-HBV cohort (2.7 %; 95 % CI, 2.5–3.0 %) (*p* < 0.001), and the aHR was 2.58 (95 % CI, 1.95-3.42; *p* < 0.001). Multivariable stratified analysis further verified significant associations of CKD with HBV in men of any age (aHR, 2.98; 95 % CI, 2.32–3.83, *p* < 0.001 for men aged <50 years; aHR, 1.58; 95 % CI, 1.31–1.91, *p* < 0.001 for men aged ≧50 years) and women under the age of 50 (aHR, 2.99; 95 % CI, 2.04–4.42, *p* < 0.001), but no significant association in women aged 50 or over.

**Conclusion:**

Untreated chronic HBV infection is associated with increased risk of CKD. Hence, high-risk HBV-infected subjects should have targeted monitoring for the development of CKD.

**Electronic supplementary material:**

The online version of this article (doi:10.1186/s12882-015-0106-5) contains supplementary material, which is available to authorized users.

## Background

Chronic kidney disease (CKD) is a global public health burden because of its increasing incidence and prevalence and progressive nature to end-stage renal disease (ESRD) [1]. Therefore, it is critical to identify risk factors for CKD to allow for effective surveillance. Beyond the traditional cardiovascular risk factors, infectious disease is an under-recognized risk factor for CKD [2].

Hepatitis B affects approximately 350 million people worldwide [3], and is associated with high mortality and morbidity [4]. Apart from major liver complications, clinical evidence suggests that chronic HBV infection has a negative impact on renal function. HBV infection can lead to glomerulonephritis, even in the absence of cirrhosis [5]. A small controlled prospective study indicated that individual estimated glomerular filtration rate declined by approximately −2 ml/min/y in 60 untreated HBV-infected patients without pre-existing renal disease, diabetes, or hypertension during the median follow-up of 24 months [6]. A 2-year cross-sectional HARPE study indicated that renal abnormalities were highly prevalent in treatment-naïve patients with chronic HBV infection [7]. A 2-year GLOBE study indicated that Telbivudine improved renal function in patients with chronic HBV infection [8]. Together, these findings provide a theoretical basis for further research of the negative impact of HBV infection on renal function.

Chronic HBV infection is associated with increased insulin resistance [9] and intensification of oxidative stress [10], and these may contribute to renal injury [11]. Therefore, it seems biologically plausible that HBV-infected subjects have increased risk for CKD. Our prior nationwide cohort study has demonstrated that untreated chronic HBV infection is associated with increased risk of ESRD [12]. However, four cross-sectional studies [9, 13–15] on the role of HBV in the development of CKD have had disparate results.

To date, there have been no cohort studies of the association of chronic HBV infection with risk of incident CKD, and it is unclear which HBV-infected patients are more likely to develop CKD. This is a crucial issue, because the management of patients with coexisting HBV infection and CKD is a challenge [16] and the global burden of HBV infection and CKD is rising [17]. Taiwan has high prevalence of CKD and HBV infection, and thus provides an ideal setting for study of the relationship of both diseases. In the present study, we conducted a 13-year nationwide cohort study to examine whether untreated chronic HBV infection is associated with increased risk of incident CKD using reimbursement claims data from the Taiwan National Health Insurance Research Database (NHIRD).

## Methods

### Database

This nationwide cohort study used outpatient and inpatient claims from the NHIRD from 1996 to 2010, which is released by the National Health Research Institutes (NHRI) for Taiwan’s National Health Insurance (NHI) Program. The NHI is a government-run, compulsory-enrolment, single-payer system that had a coverage rate of more than 99 % by the end of 2010, and codes diagnoses according to ICD-9-CM (Additional file 1: Table S1). The NHIRD lacks information on laboratory and lifestyle data and severity of the disease condition. Our previous research provided details of the NHIRD [2, 12, 18–21]. In brief, the NHIRD has detailed healthcare data of 25.68 million enrollees (99.9 % of the population) based on a random sample of all enrollees of the NHI program. There were no significant differences in age, sex, or healthcare costs between the sample group and all enrollees. The NHRI approved this study. Informed consent was not required because this is a secondary data analysis.

### Study cohorts

Here, we used methods substantially similar to those we used in our earlier paper regarding the association of HBV with ESRD [12], in which, briefly, we used the claims data from the NHIRD and identified 88790 study subjects from 1999 to 2010. In the present study, we identified all adults with chronic HBV infection between 1 January 1998 and 31 December 2010 from the NHIRD. The index date was defined on the date of chronic HBV infection claim during the entry period. We excluded subjects who had HCV infection from 1996 to 2010 and CKD/ESRD from 1996 to the index date, or took nucleos(t)ide analogues (lamivudine, adefovir, entecavir, telbivudine) [12] before CKD event occurred. The NHI program in Taiwan was initiated in 1995, so the dataset only allows tracking of medical services from 1996 to 2010. In Taiwan, the NHI program has reimbursed nucleos(t)ide analogues for patients with chronic HBV infection since October 1, 2003 [22]. Thus, we could identify HBV patients using nucleos(t)ide analogues in the NHIRD. There were 17796 untreated subjects with chronic HBV infection.

The non-HBV (control) cohort was extracted from the remaining subjects in the database. We first excluded subjects who had claim-based diagnoses of HCV or HBV infection from 1996 to 2010, or CKD/ESRD from 1996 to the index date. To consider the potential confounding by age, sex, and calendar year, we performed the individual matching technique in selecting controls. For each HBV case, we selected, by using simple random sampling method, 4 controls with the same gender and age category (18–39, 40–49, 50–59, 60–69, or ≧70 years) as the HBV case. Additionally, the eligible controls must be actively insured for at least 1 day in the year when the HBV case was identified. We also managed to exclude the situation in which one control served multiple cases. By doing so, once a beneficiary was selected in a given year during the HBV patient recruitment period (*i.e*., 1998–2010), he/she was then eliminated from the pool of potential controls, which provide reassurance that no control subjects were repeatedly selected. Thus, for controls, the index date was within the same year of the index date of their matched cases. We used age stratification because previous research indicated an age-dependent association of HBV infection [4] and of CKD [19]. The control group ultimately included 71184 subjects.

### Definition of CKD

The claims-based diagnosis of CKD was defined by the presence of 1 inpatient or 2 outpatient ICD-9-CM code 585 [23] in the claims and without catastrophic illness registration cards for ESRD (indicating the need for renal replacement therapy). The Taiwan Society of Nephrology launched a nationwide CKD Preventive Project covering CKD stages 1–5 in 2004. The simplified Modification of Diet in Renal Disease equation along with proteinuria has been used to estimate the national prevalence of 5-stage CKD in Taiwan [1]. The ICD-9-CM code 585 is consistent with the National Kidney Foundation’s Kidney Disease Outcome Quality Initiative definition of CKD stages 1–5 [19], allowing for comparisons of the incidence and prevalence of CKD in Taiwan and the United States [24]. However, the CKD stage (severity) cannot be assessed in the NHIRD.

### Main outcome measurement

Both cohorts were followed from the index date to the first diagnosis of CKD, death, or the end of 2010. Death is a competing event for any event of progression because patients who die during follow-up can no longer progress after death [25]. Therefore, death before reaching CKD, which could lead to informative censoring, was considered as a competing risk event in estimating the incidence of CKD. Death was defined by withdrawal from the NHI program [12].

### Potential confounders

We considered several confounders associated with CKD [19], including major comorbidities between 1 January 1996 and the index date (diabetes, hypertension, coronary heart disease, hyperlipidemia, and cirrhosis), number of medical visits, urbanization level (urban, suburban, and rural) and enrollee category (EC), from EC1 (highest status) to EC4 (lowest status), as proxy measures of socioeconomic status. Previous Taiwanese studies reported an association of CKD with geographic region of residence [19, 26]. Thus, geographic region of residence (northern, central, southern, or eastern Taiwan) was included to minimize potential confounding due to differences in accessibility and availability of medical care [18]. Herbs containing aristolochic acid, including (Guan) Mu Tong and (Guang) Fangchi, were associated with increased risk of CKD and could be identified from the NHIRD only before November 2003 [27]. Nevertheless, use of these herbs greater than 30 days from 1996 to endpoint outcome was also recorded. We also considered Deyo-Charlson Comorbidity Index (CCI) score and propensity score to control confounding and reduce bias in the background covariates between both cohorts in studies using healthcare administrative databases [28–30]. HBV status as the dependent variable and background covariates (including age, sex, major comorbidities, geographic region, urbanization level, EC, number of medial visits, and CCI score) as the independent variables were included in the calculation of propensity score using logistic regression. The propensity score model had a c-index of 0.66, indicating fair discrimination between the two cohorts [12].

### Statistical analyses

We calculated and compared the cumulative incidence of CKD in data with competing risk by the modified Kaplan-Meier method and Gray’s method [31], and tested differences in the full time-to-event distributions between the study cohorts using modified log-rank test. We checked the interaction terms of HBV with sex, HBV with age, and HBV with age and sex, all of which were statistically significant. After confirming the assumption of proportional hazards, we applied the modified Cox proportional hazard model in the presence of competing mortality to examine the association of HBV with CKD [32], with adjustment for all covariates (age per year, sex, major comorbidities, use of herbs containing aristolochic acid, geographic region, urbanization level, EC, number of medical visits, CCI score, and propensity score) and three significant above-mentioned interaction terms. Next, we determined the influence of HBV on CKD according to sex and age in the presence of competing mortality. For this analysis, we classified patients as younger than 50 years or as 50 years and older, because previous research indicated that steatosis is a characteristic feature of chronic HBV infection [33] and that the biggest gender difference in steatosis prevalence was observed in individuals younger than 50 years [34]. As we simultaneously included comorbidities, social characteristics, CCI and propensity score in the multivariate regression model, a potential numerical problem concerned multicollinearity between covariates, which might render estimated regression coefficients invalid. We examined the potential multicollinearity among covariates and found that multicollinearity should not be a concern in our data as the multivariate Cox regression model had no large estimated slope coefficients and estimated standard error of the mean [35]. Besides, the tolerance level was greater than 0.1 for all covariates. We analyzed all data with SAS (version 9.2; SAS Institute, Inc., Cary, N.C.) and considered a two-sided *p*-value less than 0.05 as statistically significant.

## Results

### Baseline characteristics of the study cohorts

The male-to-female ratio in the HBV cohort was 1.4. Compared with the non-HBV (control) cohort, the HBV cohort was more likely to have comorbidities including diabetes, hypertension, hyperlipidemia, and cirrhosis, as well as frequent use of herbs containing aristolochic acid, higher socioeconomic status (EC 1 and 2), more medical visits, higher propensity score and CCI score, and to reside in urban areas and northern, central, and eastern regions; all with considerable significance (Table 1).

**Table 1:** Sociodemographic characteristics and comorbidities between cohorts with and without hepatatis B virus (HBV) infection in Taiwan, 1998–2010 (*n* = 88980)

	HBV cohort	Non-HBV cohort	
Variables	(*n* = 17796) N (%)	(*n* = 71184) N (%)	*P*
Sex			1.00
Men	10428 (58.6)	41712 (58.6)	
Women	7368 (41.4)	29472 (41.4)	
Age (year)			1.00
18–39	8514 (47.8)	34056 (47.8)	
40–49	4914 (27.6)	19656 (27.6)	
50–59	2857 (16.1)	11428 (16.1)	
60–69	1146 (6.4)	4584 (6.4)	
≧ 70	365 (2.1)	1460 (2.1)	
Major comorbidities			
Diabetes	1660 (9.3)	5181 (7.3)	<0.001
Hypertension	2511 (14.1)	9020 (12.7)	<0.001
Coronary heart disease	1039 (5.8)	4392 (6.2)	0.10
Hyperlipidemia	2484 (14.0)	66.71 (9.4)	<0.001
Cirrhosis	638 (3.6)	466 (0.7)	<0.001
Use of herbs containing aristolochic acid	365 (2.1)	931 (1.3)	<0.001
Geographic region			
Northern	8967 (50.4)	35017 (49.2)	
Central	4191 (23.6)	16280 (22.9)	
Eastern	434 (2.4)	1588 (2.2)	
Southern	4204 (23.6)	18299 (25.7)	
Urbanization level			0.001
Urban	5939 (33.4)	22745 (32.0)	
Suburban	8216 (46.2)	33438 (47.0)	
Rural	3640 (20.4)	15002 (21.0)	
Enrollee category			
1	9420 (52.9)	35633 (50.0)	
2	546 (3.1)	1830 (2.6)	
3	5297 (29.8)	22157 (31.1)	
4	2533 (14.2)	11564 (16.3)	
No. of medical visits (mean ± SD)	17.7 ± 15.1	15.8 ± 15.3	<0.001
Propensity score (mean ± SD)	0.23 ± 0.09	0.19 ± 0.07	<0.001
Charlson Comorbiditv Index score (mean ± SD)	1.1 ± 1.2	0.6 ± 1.2	<0.001

Categorical variables given as number (percentage); continuous variable, as mean ± standard deviation

### Cumulative incidences of CKD between the HBV and control cohorts in the presence of competing mortality

**Fig. 1:**
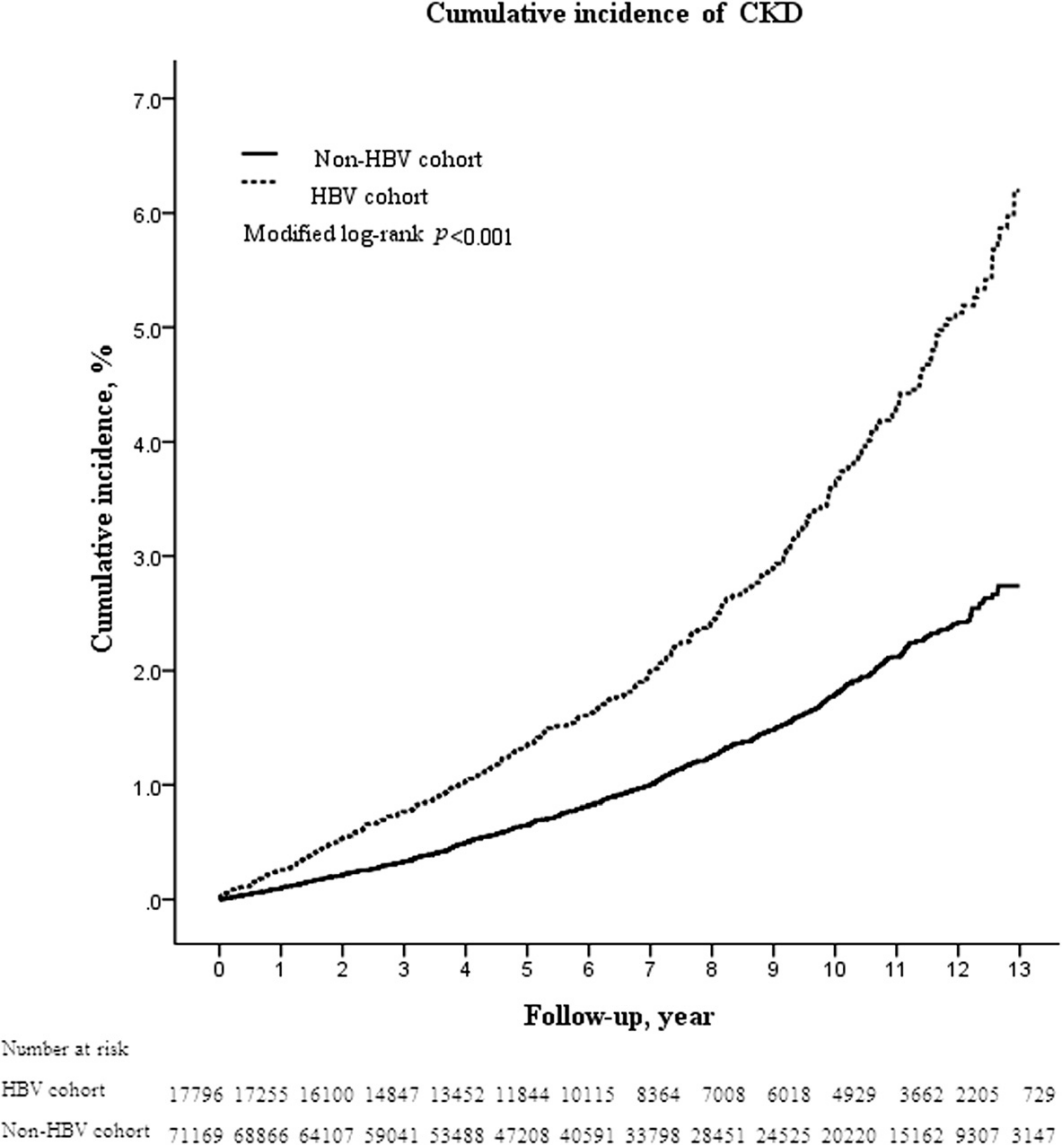
Cumulative incidence of CKD in the HBV and non-HBV cohorts. Data were compiled after adjustment for competing mortality

The 1-, 3-, 5-, 7-, 9-, 11-, and 13-year cumulative incidences of CKD were 0.26 % vs. 0.10 %, 0.77 % vs. 0.33 %, 1.35 % vs. 0.65 %, 2.00 % vs. 1.00 %, 2.90 % vs. 1.48 %, 4.30 % vs. 2.12 %, and 6.20 % vs. 2.74 %, respectively, in the HBV cohort compared with the control cohort (all *p* < 0.001, Table 2). Therefore, the risk of CKD was significantly higher in the HBV cohort (13-year cumulative incidence, 6.2 %; 95 % CI, 5.4–7.1 %) than the control cohort (2.7 %; 95 % CI, 2.5–3.0 %) (*p* < 0.001, Fig. 1).

**Table 2:** Cumulative incidences of chronic kidney disease (CKD) between the HBV and non-HBV cohorts in the presence of competing mortality

	HBV cohort, (95 % CI)	Non-HBV cohort, (95 % CI)	P*
CKD event			
At 1 year	0.26 % (0.19–0.35 %)	0.10 % (0.08–0.12 %)	<0.001
At 3 years	0.77 % (0.64–0.92 %)	0.33 % (0.29–0.38 %)	<0.001
At 5 years	1.35 % (1.17–1.55 %)	0.65 % (0.59–0.72 %)	<0.001
At 7 years	2.00 % (1.76–2.26 %)	1.00 % (0.91–1.09 %)	<0.001
At 9 year	2.90 % (2.58–3.24 %)	1.48 % (1.37–1.60 %)	<0.001
At 11 year	4.30 % (3.85–4.79 %)	2.12 % (1.96–2.29 %)	<0.001
At 13 year	6.20 % (5.39–7.08 %)	2.74 % (2.49–3.01 %)	<0.001

*Two sample proportion test. Abbreviations: *CI* confidence interval

### Chronic HBV infection as an independent risk factor for CKD

The mean duration of follow-up was 6.55 and 6.56 years for the HBV and control cohorts, respectively (Additional file 2: Table S2). Among the 88980 subjects, 1247 subjects (1.4 %) developed CKD during the study period, with 425 (2.4 %) and 822 (1.2 %) from the HBV and control cohorts, respectively. The modified multivariable-adjusted Cox proportional hazard model in the presence of competing mortality revealed that CKD was independently associated with HBV (adjusted hazard ratio [aHR], 2.58; 95 % confidence interval [CI], 1.95–3.42; *p* < 0.001), men (1.38; 1.11–1.73; *p* = 0.004); age (1.06; 1.05–1.07; *p* < 0.001), diabetes (2.41; 1.94–2.99; *p* < 0.001), hypertension (1.92; 1.64–2.24; *p* < 0.001), residence in central (1.31; 1.12–1.53; *p* < 0.001), eastern (1.49; 1.06–2.09; *p* = 0.023), southern (1.21; 1.04–1.42; *p* = 0.016) Taiwan, and the number of medical visits (1.01; 1.009–1.014; *p* < 0.001) (Table 3).

**Table 3:** Crude and adjusted hazard ratios (HR) for chronic kidney disease (CKD), with adjustment for competing morality

Variable	Crude			Adjusted^*^		
	HR	(95 % CI)	*P*	HR	(95 % CI)	*P*
HBV (Yes/No)	2.08	1.85–2.34	<0.001	2.58	1.95–3.42	<0.001
Sex (Men/Women)	1.32	1.18–1.49	<0.001	1.38	1.11–1.73	0.004
Age (per year)	1.07	1.06–1.07	<0.001	1.06	1.05–1.07	<0.001
Major comorbidities (Yes/No)						
Diabetes	5.30	4.69–5.98	<0.001	2.41	1.94–2.99	<0.001
Hypertension	5.00	4.47–5.60	<0.001	1.92	1.64–2.24	<0.001
Coronary heart disease	2.88	2.47–3.37	<0.001	0.90	0.66–1.23	0.51
Hyperlipidemia	2.77	2.41–3.18	<0.001	1.19	0.95–1.49	0.13
Cirrhosis	2.29	1.67–3.15	<0.001	1.00	0.33–3.02	0.10
Use of herbs containing aristolochic acid (Yes/No)	1.13	0.75–1.70	0.57	0.88	0.59–1.33	0.55
Geographic region						
Northern	1.00	Reference		1.00	Reference	
Central	1.22	1.08–1.39	0.002	1.31	1.12–1.53	<0.001
Eastern	1.40	1.03–1.92	0.034	1.49	1.06–2.09	0.023
Southern	1.19	1.05–1.34	0.006	1.21	1.04–1.42	0.016
Urbanization level						
Urban	1.00	Reference		1.00	Reference	
Suburban	0.94	0.84–1.05	0.27	0.98	0.85–1.14	0.82
Rural	1.39	1.23–1.58	<0.001	0.92	0.77–1.11	0.38
Enrollee category						
1	1.00	Reference		1.00	Reference	
2	0.55	0.34–0.87	0.011	0.86	0.53–1.38	0.53
3	1.52	1.36–1.70	<0.001	1.14	0.97–1.35	0.11
4	1.45	1.27–1.66	<0.001	1.12	0.91–1.38	0.28
No. of medical visits	1.02	1.01–1.02	<0.001	1.01	1.009–1.014	<0.001
Charlson Comorbidity Index score	1.31	1.29–1.34	<0.001	0.97	0.81–1.14	0.68
Propensity score	1.42	1.36–1.47	<0.001	1.10	0.77–1.57	0.60

Abbreviations: *HBV* hepatitis B virus, *CI* confidence interval

^*^Adjusted for all covariates (age per year, sex, major comorbidities, use of herbs containing aristolochic acid, geographic region, urbanization level, enrollee category, number of medical visits, Charlson Comorbidity Index score, and propensity score), and interaction terms (HBV/age, HBV/sex, and HBV/age/sex)

**Table 4:** Association of chronic kidney disease (CKD) with HBV, stratified by sex and age, with adjustment for competing mortality

	HBV cohort			Non-HBV cohort				Hazard ratio (95 % CI)		
	n	PY	CKD events	n	PY	CKD events	Crude	*P*	Adjusted^*^	*P*
Sex										
Men	10428	68249	294	41712	274693	525	2.26 (1.96–2.61)	<0.001	3.05 (2.50–3.71)†	<0.001
Women	7368	48243	131	29472	192357	297	1.77 (1.44–2.18)	<0.001	2.56 (1.93–3.41)†	<0.001
Age (year)										
< 50	13428	84358	203	53712	337813	191	4.30 (3.53–5.24)	<0.001	2.86 (1.98–4.13)	<0.001
≧50	4368	32133	222	17472	129237	631	1.40 (1.20–1.63)	<0.001	1.19 (0.90–1.56)	0.22
Men										
< 50 years	7888	49981	140	31552	200858	132	4.28 (3.38–5.43)	<0.001	2.98 (2.32–3.83)	<0.001
≧50 years	2540	18268	154	10160	73835	393	1.57 (1.30–1.89)	<0.001	1.58 (1.31–1.91)	<0.001
Women										
< 50 years	5540	34378	63	22160	136955	59	4.34 (3.05–6.19)	<0.001	2.99 (2.04–4.42)	<0.001
≧50 years	1828	13865	68	7312	55402	238	1.13 (0.86–1.48)	0.37	1.18 (0.90–1.55)	0.24

Abbreviations: *HBV* hepatitis B virus, *PY* person-year, *CI* confidence interval

*Adjusted for all covariates (age, sex, major comorbidities, use of herbs containing aristolochic acid, geographic region, urbanization level, enrollee category, number of medical visits, Charlson Comorbidity Index score, and propensity score) and interaction terms (HBV/age, HBV/sex, HBV/age/sex), minus the covariate and interaction terms on which stratified

†Age was treated as a continuous variable in multivariate analysis

We further performed a sensitivity analysis to test the robustness of these results. We excluded diabetic or cirrhotic subjects from both cohorts at baseline, also indicating a positive association of HBV with CKD (2.61, 1.97–3.46; 2.58, 2.04–3.99; respectively) (data not shown).

### Association of CKD with HBV by sex and age

After adjusting for competing mortality, multivariable stratified analysis further verified significant associations of CKD risk with HBV in men (adjusted HR, 3.05; 95 % CI, 2.50–3.71; *p* < 0.001), women (2.56; 1.93–3.41; *p* < 0.001), subjects younger than 50 years (2.86; 1.98–4.13; *p* < 0.001), men younger (2.98; 2.32–3.83; *p* < 0.001) and older than 50 years (1.58; 1.31–1.91; *p* < 0.001), and women younger than 50 years (2.99; 2.04–4.42; *p* < 0.001), and no significant association in subjects older than 50 years (1.19; 0.90–1.56; *p* = 0.22) and women older than 50 years (1.18; 0.90–1.55; *p* = 0.24) (Table 4).

## Discussion

To our knowledge, this is the first large nationwide cohort study to demonstrate an increased risk for CKD in untreated subjects with chronic HBV infection and simultaneously to determine the overall risk and age- and sex-specific risks of CKD. In this study, CKD was 2.58-fold more likely in HBV-infected subjects, relative to controls, after controlling for potential confounders and competing mortality. We also found that the risk of CKD in HBV-infected subjects was somewhat similar (range from 2 to 3) in men of any age and women under the age of 50. Noteworthily, HBV was not associated with CKD risk in women aged 50 or over. This information has important clinical implications for the design of surveillance programs that assess chronic HBV infection and CKD.

The association between chronic HBV infection and the development of CKD remains controversial. Two cross-sectional studies [7, 15] demonstrated a positive association of HBV with CKD. A 2-year multicentric cross-sectional French study reported that CKD was highly prevalent in 268 treatment-native patients with chronic HBV infection [7]. A cross-sectional Taiwanese study [15] reported that HBV was associated with CKD in 416 HBV-infected adults; however, another cross-sectional Taiwanese study by the same research group reported that HBV was not associated with CKD or proteinuria in 5424 HBV-infected adults [14]. Similarly, a cross-sectional study of 328 HBV-infected adults in Beijing [13] reported that HBV was not associated with CKD or albuminuria. On the other hand, a cross-sectional Japanese study of 130 HBV-infected adults [9] showed that HBV was not associated with albuminuria, but was inversely associated with CKD. The discrepancies among these studies may be attributed to the cross-sectional nature of their design, use of small sample sizes, and differences among the specific populations. Moreover, cross-sectional analyses cannot establish whether HBV infection preceded development of CKD. The present study was cohort design and used a large dataset, which afforded considerable statistical power and allowed tracking of incident CKD events over 13 years. We demonstrated an increased risk of CKD in untreated patients with chronic HBV infection that was independent of the presence of diabetes or cirrhosis.

Our HBV cohort tended to be younger and male, and to have a higher socioeconomic status, consistent with our prior research [12]. On stratified analysis, we found significant associations of HBV with CKD risk in men of any age (especially under the age of 50) and women under the age of 50, similar to our prior research [12]. Previous research reported that men have increased risk for development of CKD [19], and that men have a lower clearance rate than females, so are therefore more susceptible to development of chronic HBV infection [36]. Steatosis, a characteristic feature of chronic HBV infection, can increase lipid peroxidation and plasma inflammatory biomarkers [33], and these may contribute to endothelial dysfunction and renal injury [11]. The biggest sex difference in steatosis occurs in subjects younger than 50 years [33]. Taken together, these results may explain our observations regarding the effect of age and gender on the risk for development of CKD following untreated chronic HBV infection.

The pathogenesis of HBV-mediated renal injury probably depends on interactions of the virus with the host and various environmental factors [3]. Our results indicated that CKD ensued following untreated chronic HBV infection during the mean follow-up of 5.7 years, the interval of which was similar to 60 months in a case series [5]. HBV-related renal injury is believed to be due to the deposition of immune complexes of HBV antigens and host antibodies [3]. Other research also explored the possible effect of HBV on the development of nephropathy. HBV antigens (HBsAg, HBcAg, and HBeAg) and HBV DNA have been detected in glomeruli [37] and tubular epithelia [38], respectively. HBV DNA negative or positive sera of patients with chronic HBV infection promoted apoptotic damage in renal tubular cells via up-regulation of Fas gene expression, and these patients also had a higher circulating level of transforming growth factor-β, which is implicated in the potentiation of apoptosis and renal fibrosis [39]. HBV is associated with increased insulin resistance [9] and oxidative stress [10], and these are implicated in the progression of nephropathy [11]. However, only a small number of the 350 million HBV carriers develop glomerulonephritis [4]. This indicates that chronic HBV infection by itself is insufficient for development of nephropathy, and that additional socio-environmental conditions, alterations in cell-mediated immunity, genetic susceptibility, and other factors are also important [3]. This may explain the lower 13-year cumulative incidence rate (6.2 %) of CKD following untreated chronic HBV infection in this study. However, the HBV-positive population is increasing by 50 million per year, despite a marked increase in vaccination rates [40]. Therefore, it is conceivable that the burden of CKD following chronic HBV infection is increasing.

The major strength of our study is that it was designed to reduce selection bias (due to use of a large nationwide population-based and highly representative sample with random sampling), environmental effects (due to the availability of socioeconomic indicators for all subjects) [18], and detection bias (due to consideration of complete histories of the use of medical services) [19]. In addition, the study population was well defined and follow-up was complete because our design relied on computerized registries that provided complete nationwide coverage. Therefore, our finding of increased risk of CKD in HBV-infected subjects is robust.

Some potential limitations should be noted. *First*, misclassification of diseases may occur when an administration database is used. However, the NHI Administration established an audit and penalty system for quality monitoring to ensure accuracy of claims and minimize misclassification error [41]. Moreover, both CKD and viral hepatitis are important health problems in Taiwan, so the government has strict guidelines for diagnosis [42]. *Second*, some of our control subjects may have had sub-clinical HBV infection. However, if HBV is associated with CKD, this misclassification would lead to lower HRs, thus supporting the robustness of our findings. *Third*, although nucleos(t)ide analogues for HBV patients have been covered under the NHI program since October 1, 2003, this reimbursement requires patients to fulfill certain criteria, such as two-fold increase or more in serum alanine aminotransferase level and HBV DNA titer greater than 2000 IU/mL [43]. Some HBV patients may use self-paid nucleos(t)ide analogues before October 1, 2003 or when they do not fulfill reimbursed criteria, and thus may be inappropriately classified into the untreated cohort. These potential misclassifications may lead to an underestimation of the association [22]. *Fourth*, the NHIRD lacks information on family history of kidney diseases, lifestyle, body weight, and laboratory data (*e.g.,* levels of serum HBV DNA and HBV genotype), which may contribute to CKD risk. Thus, this may lead to the difference of propensity score between both cohorts. Nevertheless, we added CCI score into the propensity analysis in order to reach the comparability of both cohorts and included CCI score and propensity score in multivariable and stratified analyses to control confounding in healthcare administrative databases [12, 28, 29]. Although unmeasured confounders my still exist as with any observational study, we believe the methodology used in the present study is solid and robust.

## Conclusions

This large national cohort study indicates that untreated chronic HBV infection is associated with increased risk of CKD, especially males and younger age. High-risk HBV-infected subjects should have targeted monitoring for the development of CKD.

## Additional files

Additional file 1: Table S1. ICD-9-CM codes in the present study. Additional file 2: Table S2. Chronic kidney disease (CKD) occurrence over a 13-year follow-up.

## Abbreviations

CKD: Chronic kidney disease
HBV: Hepatitis B virus
HR: Hazard ratio
CI: Confidence interval
ESRD: End-stage renal disease
NHIRD: National Health Insurance Research Database
NHRI: National Health Research Institutes
NHI: National Health Insurance
EC: Enrollee category
CCI: Charlson Comorbidity Index
