# Supplementary material for: 13-year nationwide cohort study of chronic kidney disease risk among treatment-naïve patients with chronic hepatitis B in Taiwan
Source: BMC Nephrol. 2015 Jul 22;16:110. doi: 10.1186/s12882-015-0106-5 (PMC4508999; doi:10.1186/s12882-015-0106-5)
Supplement: Additional file 1: Table S1. — ICD-9-CM codes in the present study. [file 12882_2015_106_MOESM1_ESM.doc]

| **Additional file 1: Table S1.** ICD-9-CM codes in the present study | |
| --- | --- |
| Chronic HBV infection | 070.22, 070.23, 070.32, 070.33, V02.61 |
| HCV infection | 070.41, 070.44, 070.51, 070.54, V02.62 |
| Chronic kidney disease | 585 |
| Diabetes | 250 |
| Hypertension | 401-405 |
| Coronary heart disease | 410-414 |
| Hyperlipidemia | 272-272.4 |
| Cirrhosis | 571.2, 571.5, 571.6 |
