# Supplementary material for: 13-year nationwide cohort study of chronic kidney disease risk among treatment-naïve patients with chronic hepatitis B in Taiwan
Source: BMC Nephrol. 2015 Jul 22;16:110. doi: 10.1186/s12882-015-0106-5 (PMC4508999; doi:10.1186/s12882-015-0106-5)
Supplement: Additional file 2: Table S2. — Chronic kidney disease (CKD) occurrence over a 13-year follow-up. [file 12882_2015_106_MOESM2_ESM.doc]

| **Additional file 2: Table S2.** Chronic kidney disease (CKD) occurrence over a 13-year follow-up | | | |
| --- | --- | --- | --- |
|  | Mean follow-up (y) | Total follow-up (PY) | CKD events (%) |
| HBV cohort (n=17796) | 6.55 | 467050 | 425 (2.4) |
| Non-HBV cohort (n=71184) | 6.56 | 116491 | 822 (1.2) |

Abbreviations: HBV, hepatitis B virus; y, year; PY, person-year.
